# Performance Evaluation of UAV-Enabled LoRa Networks for Disaster Management Applications

**DOI:** 10.3390/s20082396

**Published:** 2020-04-23

**Authors:** Omar A. Saraereh, Amer Alsaraira, Imran Khan, Peerapong Uthansakul

**Affiliations:** 1 Department of Electrical Engineering, Hashemite University, Zarqa 13133, Jordan; eloas2@hu.edu.jo; 2 College of Engineering and Technology, American University of the Middle East, Kuwait; Amer.Alsaraira@aum.edu.kw; 3 Department of Electrical Engineering, University of Engineering and Technology Peshawar, P.O.B. 814 KPK, Pakistan; ikn.eup121@gmail.com; 4 School of Telecommunication Engineering, Suranaree University of Technology, Nakhon Ratchasima 30000, Thailand

**Keywords:** LoRA, packet reception rate, UAV, topology control

## Abstract

In hostile and remote environments, such as mountains, forests or suburban areas, traditional communications may not be available, especially after a disaster, such as a flood, a forest fire or an earthquake. In these situations, the wireless networks may become congested or completely disrupted and may not be adequate to support the traffic generated by rescuers. It is also considered as the key tool in Corona Virus (COVID-19) battle. Moreover, the conventional approaches with fixed gateways may not work either, and this might lead to decoding errors due to the large distance between mobile nodes and the gateway. To avoid the decoding errors and improve the reliability of the messages, we propose to use intermediate Unmanned Aerial Vehicles (UAVs) to transfer messages from ground-based Long Range (LoRa) nodes to the remote base station (BS). Specifically, this UAV-enabled LoRa architecture is based on the ad hoc WiFi network, wherein, UAVs act as relays for the traffic generated between LoRa nodes and BS. To make the architecture more efficient, a distributed topology control algorithm is also proposed for UAVs. The algorithm is based on virtual spring forces and movement prediction technique that periodically updates the UAV topology to adapt to the movement of the ground-based LoRa nodes that move on the surface. The simulation results show the feasibility of the proposed approach for packet reception rate and average delay quality of service (QoS) metrics. It is observed that the mechanisms implemented in a UAV-enabled LoRa network effectively help to improve the packet reception rate with nominal buffer delays.

## 1. Introduction

The ubiquity of the Internet, new and innovative communication protocols and the miniaturization of computational devices gave rise to a new paradigm called the Internet-of-Things (IoT) [1]. The number of IoT devices is projected to grow steadily in the years to come, especially thanks to their possible applications in a vast and diverse range of fields. IoT solutions are being used in the industry, in agriculture, in smart cities and in many other sectors [2,3]. In many cases, IoT devices are battery-powered and subject to very strict power constraints. For this reason, a new range of low power wireless communication protocols has been developed and standardized to support the operation of Low Power Wide Area Networks (LPWANs). These networks are typically formed by inexpensive, simple devices that need to communicate infrequently over long distances at low bit-rates. Long Range (LoRa) Wide Area Network (WAN) is one of the most promising technologies enabling LPWANs [4,5]. LoRaWAN takes advantage of Spread Spectrum (SS), chirp orthogonality and the good propagation characteristics of the sub-GHz spectrum to provide reliable communication over long distances. This comes at the expense of the bit rate and of the maximum time interval between consecutive transmissions due to duty cycle limitations in the bands used by the protocol.

Unmanned Aerial Vehicles (UAV), commonly called drones, are flying vehicles that can operate without the need of a remote pilot. Exclusive prerogative of the military for many years, UAVs are now commercially available and their relatively low price makes them appealing for a wide variety of applications in a diverse range of scenarios [6,7,8,9]. The price range can vary between 20$–300$ for small battery-powered Radio Frequency (RF) drones such as the Parrot Bebop 2, to more than 3000$ for professional drones with advanced control systems and sensors such as the DJI Spreading Wings S1000. In the consumer market segment, drones are mainly used for taking aerial pictures and videos, while in the commercial segment the range of applications is more diverse: mapping of geographical areas, product delivery, and logistics, data collection, crop monitoring, and surveillance [10,11,12]. Drones are increasingly being used also in applications such as crime prevention, weather and meteorology, search and rescue operations, border and maritime patrol and forest fire monitoring. In case of a disaster, such as an earthquake, a flood or a wildfire [13], UAVs can be used to support the operations of the rescuers, to localize the victims and to create a backhaul network in case of absence or disruption of the communication facilities [14]. In this last circumstance, the relay network formed by UAVs can support the data generated by isolated IoT devices that have been deployed in advance or during the rescue operations. During the ongoing pandemic circumstances, the UAVs can be deployed against the Corona Virus (COVID-19) battle. Some of the important applications of UAVs against corona are virtual tours of quarantined regions, sending medical supplied to patients, hospital and areas to which the access is difficult, prevent virus spread via virtual and wireless control.

### 1.1. Related Work

Petajajarvi et al. [15] conducted empirical measures to determine the maximum range of LoRa in a small rural city in Finland. The results show that, by using the maximum Spread Factor (SF) and a bandwidth of 125 kHz in the 868 MHz band, the signal can reach the gateway from a 15 km distance on the ground and 30 km on water, with increments of the Packet Drop Rate (PDR) proportional to the distance. The authors of [16] have performed a similar analysis in a medium-density urban area, noting in this case, that the maximum range under the same conditions described in [15] is only 5.8 km, mainly due to the presence of obstacles in the line-of-sight. Moheddine et al. in [17] proposed the concept of flying gateways for enabling communication between IoT and UAV networks. Though their work used Raspberry Pi boards, they expected to improve the performance of the network using LoRa devices.

In [18], the signal reception in presence of indoor end devices is also evaluated. The authors report that at a distance of 1.2 km from the gateway the PDR is already between 60% and 90%. The authors conclude that the presence of line-of-sight is particularly important and that LoRa is not suited for indoor environments if long-range and low PDR is required. The importance of line-of-sight is also outlined by Iova et al. [19]. In this case, the experiments were conducted in a forest environment in the presence of numerous trees between the transmitter and the receiver. In the experiment, the transmitter was moved further and further away from the receiver and at around 90–100 m the signal was completely lost. The authors conclude that vegetation has a huge impact on LoRa signal propagation, in fact dropping the maximum achievable range by an order of magnitude. The use of the chirp spread spectrum, a variation of Direct Sequence Spread Spectrum (DSSS), makes the LoRa modulation very resilient to noise. This assumption is verified by Angrisani et al. [20]. In the experiment, different levels of white Gaussian noise are applied to the signal starting with a minimum signal-to-noise ratio of −10 dBm. As expected, the use of a smaller bandwidth and, more significantly, the use of a higher SF improves the performance of the modulation. The coding rate, on the other hand, has a less significant impact on the performance in noisy environments.

Magrin et al. in [21] developed a module that models a subset of functionalities of the LoRaWAN physical and MAC layers. Each end device has an associated class that models the behavior of a LoRa chip and tracks its state (standby, sleep, transmission or reception). The only difference is in the fact that their proposed scheme defines 8 parallel reception paths, each one of them able to lock on an incoming packet on a given frequency. Their results show considerable improvement using the developed LoRa module. Van den Abeele et al. in [22] proposed a model similar to the one previously described. The main difference resides in the way in which inter-device interference is computed. In this case, instead of using a signal-to-interference matrix, the authors performed a series of complex MATLAB baseband simulations to measure the Bit Error Rate (BER) of a set of LoRa configurations over a white Gaussian noise channel. The BER points are then used to determine the BER curve that is then used to decide if a packet is lost for interference or not.

Goddemeier et al. [23] consider a distributed decision approach to maintain a coherent mesh network of UAVs with the objective of exploring a 3D area. The swarm has also the additional requirement of keeping the connectivity to a ground base station. Two scenarios are studied by the authors: one in which the connectivity to the base station is permanent and one in which some disconnections are allowed for the purpose of extending the exploration. Di Felice et al. [24] propose a distributed approach similar to [23], but based on VSFs. The UAV mesh network described in the paper is used to give coverage to a set of partitioned ground nodes. The least visited cell in the visibility zone is then selected for exploration and an attractive spring force is computed between the UAV and the center of the cell. Chandrashekar et al. [25] study the problem of determining the minimum number of UAVs necessary to provide full coverage to clusters of isolated ground nodes and at the same time maintain connectivity between UAVs. The position of the UAV that covers this subset of clusters (supercluster) is determined by means of a convex minimization problem. The UAV to send to the new computed location is chosen by using a metric that minimizes the total travel distance of UAVs. Caillouet et al. [26] tackle the more challenging problem of optimally positioning the minimum number of UAVs, taken from the set of all available UAVs, in order to cover a set of targets on the ground, while maintaining the connectivity of the aerial mesh to a ground base station. An evaluation of the model performed by the authors proves that the algorithm always finds an optimal solution in a reasonable amount of time (1000 s). The authors also state that the impact of the connectivity constraint more than doubles, in some circumstances, the number of deployed UAVs.

Some other recent studies [27,28,29,30] also investigate the compatibility of UAV and LoRa network for enabling IoT applications. However, one of the main requirements of an LPWAN is its ability to scale to hundreds or even thousands of devices. Early attempts to assessing the scalability of LoRaWAN were performed by resorting to mathematical models. Mikhaylov et al. [31] assessed the scalability of a LoRa network in the presence of a single gateway. The authors modeled the LoRaWAN MAC as pure ALOHA and concluded that LoRaWAN can potentially scale to millions of devices in case of end devices generating only intermittent uplink traffic, but most of the devices need to be positioned very close to the gateway. The authors of [22] provided analysis for LoRaWAN. They performed simulation in ns-3 and analyzed the scalability and reliability of such networks. The authors of [32] performed measurements for received signal strength (RSS) for a LoRa network for indoor, suburban and urban environments. They concluded that for vertical transmitting antennas, the radiation pattern of UAV results in stronger RSS levels at the LoRa network.

### 1.2. Motivation and Contribution

The integration of UAVs and LPWAN protocols in disaster scenarios may offer a new cost-effective and energy-efficient way of tackling many problems arising during the operations of the rescuers. For instance, consider a forest in which a wildfire has been detected. Wildfires generally represent a worldwide problem, but America and Europe are the two continents where the incidence of forest fires is higher. The costs, in terms of economic losses and human deaths, are also considerable [13]. Wildfires typically affect rural or suburban areas where the network coverage of traditional communication networks (e.g., cellular networks) is scarce, intermittent or completely lacking. This situation is worsened by the huge propagation loss introduced by foliage and trees in the signal propagation path. In this situation UAVs can be used to establish a mesh network acting as a relay between the Base Station (BS) installed in the command post, the place where operations are managed, and the sensors carried by firemen, therefore providing more precise situational awareness to the rescuers. The mobility of the firefighters represents another huge challenge since the UAVs mesh network needs to adjust its position while maintaining the connectivity to the BS and to firemen on the ground. Unfortunately, the related literature on such works is still scarce, leaving many issues open to further investigation. In this work, our objective is to design and evaluate a UAV mesh network system, called UAV-enabled LoRa network. In this regard, the distinguishing aspects of our work and a brief comparison with the state-of-the-art solutions are given in Table 1. The main contribution of this work can be summarized as follows:

- A novel UAV-enabled LoRa network architecture is proposed for the disaster management network. The proposed solution is easy to deploy and does not incur much cost for the network.
- A decentralized topology control algorithm for the UAVs has been provided. The proposed topology control algorithm allows UAVs to preserve connectivity with the BS, despite the mobility of the Ground Nodes (GNs).
- Exhaustive performance evaluation of the UAV-enabled LoRa network has been performed in ns-3. The selected QoS metrics such as average packet reception rate, average buffered packets, and total delay have been used to assess the performance of the network.

### 1.3. Organization

The rest of the paper is organized as follows. Section 2 provides the details of the network architecture. In Section 3, details of the mobility algorithms are presented. Section 4 provides the performance assessment results along with relevant discussion. Section 5, finally, concludes this work and highlights some future research directions.

## 2. Network Setup and Architecture

**Figure 1:**
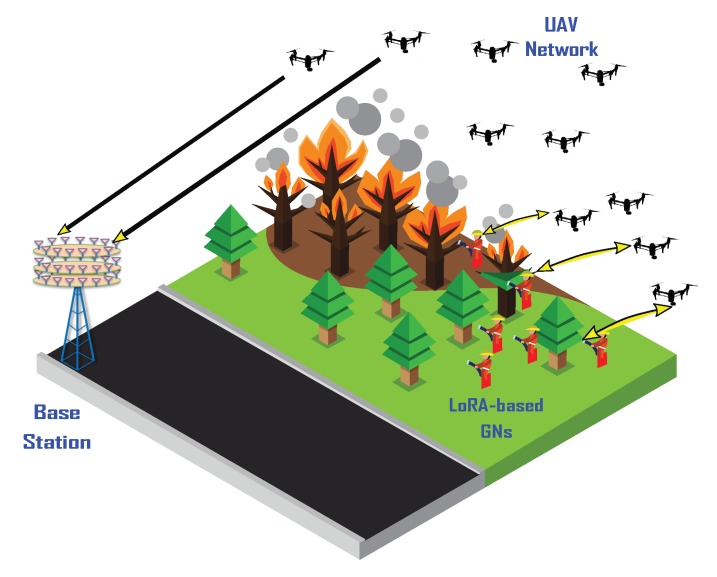
Illustration of UAV-enabled LoRa architecture in the backdrop of a wildfire.

In this section, the UAV-enabled LoRa system components and the main design choices are presented and described. The main objective of the UAV-enabled LoRa system is to provide network coverage to a set of mobile GNs using a fleet of UAVs, whose purpose is to promptly relay the collected data to a base station through an ad hoc network established between its members, as shown in Figure 1. To meet these requirements, each UAV must be able to automatically adjust its position to reflect the continuously changing topology of the GN and at the same time, it must be able to maintain a communication path with the base station. Disconnections, although difficult to avoid, must be minimized and a recovery technique must be set up to recover the connectivity whenever it is necessary. UAV-enabled LoRa network provides mechanisms to deal with all the aforementioned problems. This kind of system might be useful in a wide variety of situations, especially when there is the need to rapidly deploy a network to collect data generated by moving targets in remote or disrupted areas, where other networks might be unavailable, damaged or highly congested [38,39].

The architecture of the UAV-enabled LoRa network consists of a two-layer system, in which the first layer is composed of GNs that transmit data using LoRaWAN and the second layer is composed of a swarm of drones communicating over a WiFi ad hoc network and acting as relays between the GNs and a BS. The main components are listed and described below.

### 2.1. Ground Nodes

**Figure 2:**
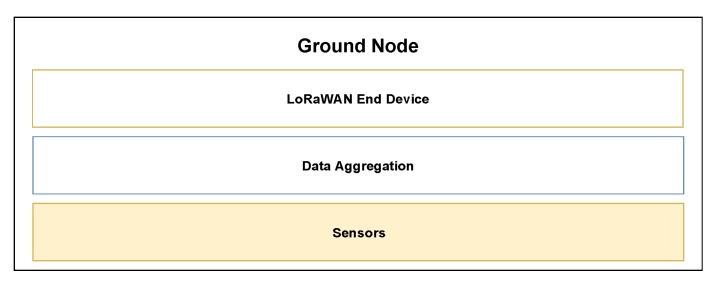
System stack of Ground Nodes (GNs).

As illustrated in Figure 2, the GNs are equipped with devices that can aggregate data coming from various sensors and transmit it using a LoRa module implementing the LoRaWAN protocol. LoRa is a proprietary modulation technique based on the chirp spread spectrum originally developed by Cycleo and then acquired by Semtech in 2012. Like many other LPWAN technologies, LoRa uses unlicensed sub-GHz bands, therefore taking advantage of the good propagation properties of the spectrum. Since the LoRa modulation is proprietary, no official comprehensive description of the modulation exists as of today, but some attempts to reverse engineer the protocol have been made. The description of the LoRa physical layer given in the present work is partially based on the little information given in the official LoRaWAN standard and on Semtech technical documents.

### 2.2. UAV Functionality

The drones are the most complex elements in the system, given the variety of tasks they need to perform. First of all, each UAV must be equipped with all the necessary sensors, controls and software for navigation and stabilization. Typical sensors include altimeters, speed meters, accelerometers, and tilt sensors. The details of the navigation and control system are outside the scope of the paper and it is simply assumed, realistically, that drones already come with all the necessary components and with a software API to interact with them. The UAVs are able to hover over a specific location or target while the specific type of engine and fuel for UAV depends on the application and is outside the scope of this work.

**Figure 3:**
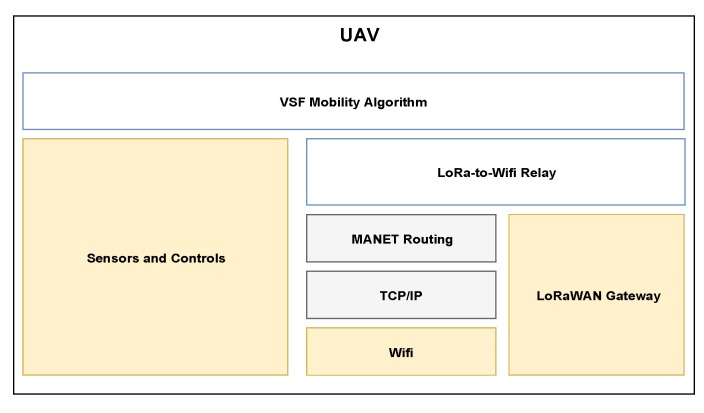
System Stack of UAV.

As shown in Figure 3, the end-to-end packet delivery over WiFi is accomplished by resorting to a traditional TCP/IP stack, with the assistance of a Mobile Ad hoc Network (MANET) routing protocol. Even though any MANET routing protocol can be used, in the current implementation of the system some mechanisms rely on the knowledge of the whole topology of the network, therefore a table-driven link-state protocol like Optimized Link State Routing (OLSR) protocol becomes necessary for the correct operation of UAV-enabled LoRa network. The module contains also a LoRa-to-WiFi relay application, responsible for adapting uplink LoRa packets received by the gateway and send them over the WiFi ad hoc network to the intended destination. The most important and complex part of the whole system is the virtual spring force (VSF) distributed mobility algorithm. This algorithm is responsible for planning the movements of the drone according to VSF computed based on parameters collected from positioning sensors, routing tables and messages exchanged with GNs and other UAVs. Given the amount of information needed, the algorithm needs to interface with almost all the previously described components to work properly.

### 2.3. Base Station

**Figure 4:**
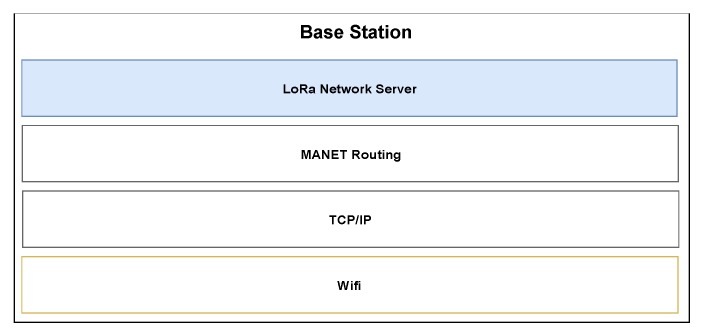
System stack of base station (BS).

In its most minimal configuration, the BS contains the same WiFi equipment of a UAV, runs the same MANET routing protocol and integrates a LoRaWAN network server that is used to manage the LoRaWAN network, as given in Figure 4.

It is considered that all UAVs fly at the same altitude in a 2D plane. A three-dimensional configuration of UAVs is still allowed in the developed system, but the benefits in terms of extended coverage to GNs and collision avoidance are not investigated as part of this work. This means that even if UAVs have different altitudes, the mobility algorithm behaves as if they are on the same plane. An altitude optimization procedure may be introduced seamlessly on top of the system at a later time. Moreover, all UAVs can move with constant speed in any direction. Drones are complex systems that require complex controllers to manage speed, acceleration, stability and heading along the six-axis. This work focuses on the performance of the networks and on the ability of the UAVs mobility algorithm to improve the coverage of GNs. UAVs also have access to the received power of neighboring UAVs and GNs or can estimate it based on their position. Routing messages can be used to collect the received power, which is necessary to determine the link budget of the links with neighboring UAVs. The received power of GNs can be collected when an uplink message is received. Eventually, the received power can be estimated by resorting to propagation models and the positions of the nodes.

## 3. Proposed UAV Topology Control Algorithm

The mobility of the drones is essential to keep the system functional and to adapt the formation to the movements of GNs. UAV-enabled LoRa network implements three mechanisms that together control the mobility behavior of the UAVs: the VSF mobility algorithm, the Connection Recovery, and Maintenance (CRM) algorithm and the Movement Prediction (MP) algorithm. These mechanisms are described in detail in the following sections.

### 3.1. Virtual Spring Force (VSF) Mobility

UAV-enabled LoRa network takes advantage of a distributed mobility algorithm through which the direction of movement is determined at each time step by a VSF that can push or pull a UAV closer or further to other UAVs or GNs. In this way, UAVs are kept in the range of each other, possible collisions are avoided and the objective of covering the GNs is pursued. In its most simple form, the algorithm runs at fixed regular intervals ΔT. At each time step, each UAV generates a set of virtual springs, having one end attached to the UAV that generated them and the other end attached to either a GN (AtG springs) or another UAV (AtA springs). For each spring, the attractive or repulsive force $F_{ij}$ between node *i* and node *j* is given by

(1) $$\begin{array}{c} F_{ij}=K\cdot \left(LB_{ij}-LB_{req}\right)x, \end{array}$$

where *K* is a constant that depends on the nature of the spring, $LB_{ij}$ is the real or estimated link budget between node *i* and node *j* and $LB_{req}$ is the required link budget. Choosing link budget measurements instead of physical distances is more meaningful for a communication system. The link budget gives a more direct indication of the quality of the link, e.g., $LB_{req}$ based on application-specific QoS requirements. The total force $F_{tot}^{i}$ applied to UAV *i* is given by

(2) $$\begin{array}{c} F_{tot}^{i}=\sum_{j=0}^{N}F_{ij} \end{array}$$

where *N* is the number of springs originating from *i*. AtG springs are established with all the GNs that at least sent one message containing their position in the last AtG seconds to *i*-th UAV. AtA springs are established with any one-hop neighboring UAV, whereby, the neighboring UAVs are selected based on the RSS levels of the connection between the UAVs. AtA and AtG springs are therefore only established with direct neighbors, thus, limiting the complexity of the interactions and the number of computations.

The *K* parameter is fundamental to determine the weight, and therefore the priority, of each spring force which is updated as

(3) $$\begin{array}{c} K_{AtA}=K_{p}\left(\frac{N_{neighs}^{\max}}{n_{neighs}}\right) \end{array}$$

where $K_{p}$ is the scaling factor, $N_{neighs}^{\max}$ is the maximum number of neighbors, and $n_{neighs}$ represents the current number of neighboring UAVs. It is worth mentioning that the value of $N_{neighs}^{\max}$ is chosen arbitrarily and must be optimized on a trial-and-error basis, yet a good value of $N_{neighs}^{\max}$ resides mostly between 6 to 8 because the UAVs tend to arrange themselves in a hexagonal grid formation. However, for different UAV in the system, $N_{neighs}^{\max}$ can be approximated with the total number of UAVs currently operating in the system. The above equation allows scaling the magnitude of the attractive/repulsive AtA forces based on the relative number of neighboring UAVs. In this way, a UAV with few neighbors will have a higher tendency to stick to the formation, while a UAV with many neighbors will exhibit the opposite behavior. This gives more importance to AtG springs when a UAV has a high number of neighboring UAVs.

The *K* parameter takes a different value at each time step for each AtG spring connected to GN *j* according to the expression

(4) $$\begin{array}{c} K_{AtG}=\frac{u_{\max}}{u_{j}} \end{array}$$

where $u_{\max}$ is the highest number of neighboring UAVs covering one or more GNs that are also covered by the current UAV and $u_{j}$ is the current number of UAVs covering GN *j*. This relation allows giving more priority to the GNs that are covered by fewer UAVs. Drones are therefore expected to distribute themselves uniformly between the GNs that are in range. This is especially useful when a group of GNs splits and the covering UAVs must decide which GNs to follow.

### 3.2. Connection Recovery and Maintenance (CRM)

Even though attractive forces already provide a cohesion mechanism that helps to keep all the members of the swarm in a compact formation, in a system like UAV-enabled LoRa network clustering is difficult to avoid. Disconnections might be caused by a series of factors, like variable environmental conditions or simply by a UAV or a group of UAVs that goes too far from nearby UAVs due to a particularly strong attractive force. In these circumstances, some mechanisms need to be integrated into the system to recover the connectivity with the UAV formation and therefore with the BS. When the connection to the BS is recovered, a connection maintenance procedure must be triggered to avoid further disconnections.

More specifically, three mobility behaviors are implemented in the system:

1. VSF mobility
2. Network Recovery Mobility (NRM)
3. Stationary Mobility (SM)

The selection of the right mobility is based on the value of two parameters: pause and persist. The pause parameter is a positive integer number that specifies the number of ΔT time intervals during which the UAV must remain stationary. When a connection with the BS is re-established thanks to the NRM, the SM is activated and kept active until, after pause×ΔT time intervals, the pause parameter reaches the value zero. This mechanism has the purpose of keeping the UAV in a stable position for some time and avoid further disconnections. The value of pause is given as

(5) $$\begin{array}{c} pause=\lfloor P_{\max}\times \frac{R_{\max}}{D_{BS}}\rfloor , \end{array}$$

where $P_{max}$ is the maximum number of pause intervals, $R_{max}$ is the maximum range of the drone and DBS is the current distance from the BS. $R_{max}$ is progressively computed by resorting to the position information periodically exchanged with the neighboring UAVs. The above expression assigns a more conservative behavior to drones that are closer to the BS and, therefore, the ones that are more likely to cause the disconnection of a big part of the network. On the contrary, peripheral UAVs can move more freely, since it is less likely that their disconnection causes the partition of other parts of the network. The pause parameter is ignored only in one circumstance, that is when the load parameter is zero. This parameter measures the number of relayed packets during a time window of fixed length $t_{load}$ and it can, therefore, be used to select those UAVs that have been inactive for some time. Since those UAVs are not useful for the operation of the system, they can keep moving according to the VSF mobility without the constraints imposed by the CRM algorithm.

The persist parameter is an integer number expressing the number of ΔT time intervals during which the drone must keep using the VSF mobility even if a connection to the BS is not available. This behavior is necessary to relax the conditions that trigger the NRM, in the hope that a path to the BS can be re-established thanks to the rearrangement of other UAVs, typically located closer to the BS. In the present work, the persist parameter is set according to the following expression

(6) $$\begin{array}{c} persist=\lfloor \frac{\left(hops-1\right)D_{BS}}{R_{\max}}\rfloor , \end{array}$$

where hops are the number of hops that were separating the UAV from the BS the last time the path was active. The above expression allows peripheral UAVs to be dragged by VSF forces for a longer time, even if a path to the BS is not available. On the contrary, UAVs closer to the BS is less tolerant of disconnections and try to recover a connection sooner than their peripheral counterparts. In normal circumstances, persist is decremented by one unit at each time step, until zero is reached. However, there is a situation in which the persist counter is updated differently. It is possible that a group of UAVs, covering almost the same set of GNs, gets separated from the rest of the swarm. The typical persist behavior would not, in this case, exploit the redundant UAVs to recover the connectivity. Instead, all the UAVs would trigger the NRM almost at the same time and all would lose the connection with the covered GNs.

In order to avoid this kind of situation, UAVs that are covering a set of GNs determine, immediately after losing the connection to the BS, the neighboring UAV with which they share the highest percentage of GNs. If this percentage is above a certain threshold of $p_{shared}$, one of the two UAVs must be redundant. Every UAV has a fixed ID that has been assigned before the operation. The UAV with the lowest ID among the two proceed to cut by half its persist intervals, while the other UAV double their persist intervals. During the next time steps, one of the redundant UAVs, the one with the lowest value of persist, triggers the NRM before the others and reposition itself closer to the BS. This return, in some cases, helps to re-establish a connection between partitioned UAVs and the BS.

### 3.3. Movement Prediction (MP)

During the operations, some GNs might go out of the range of the UAV swarm and become isolated. A recovery mechanism must, therefore, be implemented. The solution presented here is based on a combination of virtual forces and movement prediction. Using the same virtual forces that are used for the normal UAV’s mobility, allows us to easily integrate the solution in the system and at the same time, it allows us to preserve the simplicity of the distributed approach that distinguishes UAV-enabled LoRa network.

The recovery mechanism is centered around elements called holograms, whose purpose is to signal to the members of the swarm the presumed position of a lost GN or group of GNs. In practice, a hologram is simply a piece of information that in its most simple form contains a pair of spatial coordinates that can be used as a reference to compute a virtual AtG force between the hologram itself and one or more UAVs. For the system to be effective, holograms must be placed in convenient positions. Two alternatives have been considered:

- Placing holograms in correspondence of the last known position of a GN or a group of GNs
- Placing holograms in the predicted position of a GN or a group of GNs.

Since GNs are continuously moving, having only the information about their last known position is not enough to generate a useful hologram. While drones adjust their position, GNs in the meanwhile may have moved somewhere else. If we can assume that GNs keep a regular direction and speed for some time, it is possible to predict the future position of GNs from their previous recorded positions and update the hologram location accordingly at each time step. The prediction implemented in the UAV-enabled LoRa network is based on simple kinematic equations. If we consider a GN *i* at time *t*, its position $x_{i}$ and $y_{i}$ at time t+1 is given, respectively, as

(7) $$\begin{array}{cc} x_{i(t+1)}^{p} & =x_{it}+v_{it}^{x}+\frac{1}{2}a_{it}^{x}\cdot \Delta t \end{array}$$

(8) $$\begin{array}{cc} y_{i(t+1)}^{p} & =y_{it}+v_{it}^{y}+\frac{1}{2}a_{it}^{y}\cdot \Delta t \end{array}$$

where $v_{it}$ and $a_{it}$ are respectively the velocity at time *t* and the expected variation of velocity between *t* and t+1 (acceleration). The computation of all the necessary variables only requires the knowledge of the three most recent positions of a node, so that

(9) $$\begin{array}{cc} v_{it}^{x} & =x_{it}-x_{i(t-1)} \end{array}$$

(10) $$\begin{array}{cc} v_{i(t-1)}^{x} & =x_{i(t-1)}-x_{i(t-2)} \end{array}$$

(11) $$\begin{array}{cc} v_{i(t-2)}^{x} & =x_{i(t-2)}-x_{i(t-3)} \end{array}$$

where $a_{it}^{x}=a_{i(t-1)}^{x}+\Delta a_{i(t-1)}^{x}\times \Delta t$, $\Delta a_{i(t-1)}^{x}=\frac{a_{i(t-1)}^{x}-a_{i(t-2)}^{x}}{\Delta t}$, $\Delta a_{i(t-2)}^{x}=\frac{v_{i(t-1)}^{x}-v_{i(t-2)}^{x}}{\Delta t}$, $a_{i(t-1)}^{x}=\frac{v_{it}^{x}-v_{i(t-1)}^{x}}{\Delta t}$, and $v_{i(t+1)}^{x}=v_{it}^{x}+a_{it}^{x}\times \Delta t$. It is also worth mentioning that the same applies to *y* coordinates. To simplify the problem, in the present work only GNs with constant velocity are considered, so that in the previous equations the acceleration can be set to zero and only the two most recent positions are needed to perform a prediction.

Each UAV is responsible for keeping track of the GNs it has lost communication with and to send to the UAVs in proximity the information needed to predict the positions of the holograms. This information is included in a message called token and sent in unicast to all the UAVs that have an entry in the routing table. A token includes necessarily the last known position of a GN, its velocity vector and the time at which the last known position was recorded. Upon reception of a token, a hologram is created and an expiration time $t_{exp}$ is attached to it. To avoid the disruption of local AtA and AtG forces, only UAVs that are inactive (load = 0) are influenced by forces produced by holograms.

To reduce the amount of exchanged tokens, lost GNs can be first clustered in groups based on their position using a clustering algorithm. In the present work, *k*-means is used for clustering and *k*-means++ is used to better initialize the centroids and speed up the convergence. The *k*-means algorithm accepts as input the vector data that has to be clustered and the number *k* of desired clusters. *k* centroids are initialized and all data points are then associated with the closest centroid using a suitable distance measure (the Euclidean distance in our case). The mean of all the points is used as a new centroid in the next iteration of the algorithm and the said process is repeated until convergence. One of the main disadvantages of *k*-means is that it needs to know from the beginning the number *k* of clusters. The selection of *k* is a nontrivial problem that is still at the center of many types of research. In the UAV-enabled LoRa network, the *k*-means algorithm is run multiple times with different values of *k*, with *k* ranging from 2 to $k_{max}$ and the quality of the clustering is then assessed by using the average silhouette index. This index measures the similarity among members of the same cluster and their dissimilarity from the members of other clusters. In formulas, the silhouette s(i) of the data point *i* is

(12) $$\begin{array}{c} s\left(i\right)=\frac{b(i)-a(i)}{\max\{a(i)\times b(i)\}}, \end{array}$$

where a(i) is the average distance of *i* from the members of its cluster and b(i) is the lowest average distance of *i* from the members of neighboring clusters. The index values range from −1 to 1, with values close to 1 indicating that the data point *i* belongs to a good cluster and values close to −1 indicating that the data point *i* probably should belong to another cluster. To avoid having too many clusters, data points belonging to a cluster with only one point have a silhouette value of 0.

The average $s_{avg}$ of all s(i) is used to assess the overall quality of the clustering. In fact a particular clustering is only accepted if $s_{avg}$ is above a threshold $s_{thr}$. If no clustering configuration that satisfies the criteria is found, then all lost GNs are considered as members of a single cluster. After the appropriate clustering is found, a token is created for each cluster and sent to the other UAVs. The token contains the center of mass computed from the last recorded positions of the GNs belonging to the cluster, the average velocity of the cluster computed as the average of the velocity vectors associated with each GN belonging to the cluster and the time associated with the most recent recorded position among the GNs in the cluster.

## 4. Performance Evaluation

The UAV-enabled LoRa system performance is evaluated through a series of simulations performed with the ns-3 network simulator. ns-3 has been chosen over other network simulators for its modular architecture, its speed, its active community and, more importantly, for the support of all the protocols that are necessary for UAV-enabled LoRa, including a WiFi module, MANET modules and external modules for simulating LoRaWAN networks. Additional modules, utility classes, and applications have been developed specifically for implementing the missing features, such as the VSF based mobility algorithm.

The developed simulation consists of two separate channels: an AtA channel for the communications between UAVs over WiFi and an AtG channel for the communications between GNs and UAVs through LoRaWAN. Both channels are configured to use the constant speed propagation delay model so that signals propagate with a constant speed equal to the speed of light. This is a reasonable assumption, considering that signals propagate in a low-density air medium. A different path loss has instead been chosen for each channel, to better characterize two extremely different propagation conditions: AtA links are modeled with a Friis propagation model and AtG links with a Log Distance propagation model.

The WiFi ad hoc network operates using IEEE 802.11 g, a protocol that is broadly supported and implemented in cheap hardware. The modulation is ERP-OFDM with a maximum throughput of 12 Mbps, which is high enough to support the traffic generated by LoRaWAN GNs. Moreover, the selected modulation is a good compromise in terms of signal range and throughput. The transmission power and receiver sensitivity are set to the default ns-3 values, which correspond to common WiFi settings. The MANET routing protocol is OLSR. Proactively building the routing tables is, in fact, essential for the correct functioning of the UAV-enabled LoRa network mobility algorithm, since the information they contain is used to compute various important parameters. The impact of the routing protocol on the performance of the system has not been investigated as part of this work, but it is reasonable to expect that, at least in highly mobile networks, any protocol that provides fast convergence of routes and frequent periodic table updates would benefit the UAV-enabled LoRa system. The simulation parameters and their values are provided in Table 2. Note that the altitude of UAVs is set to 60 m above the ground, which corresponds to the maximum height of trees in temperate climates. This height is suitable for disaster-ridden areas where the taller infrastructures rarely exist. The terrain is considered flat and the GNs moves at a normal walking speed of 5 km/h which is commonly used in the literature [40].

**Figure 5:**
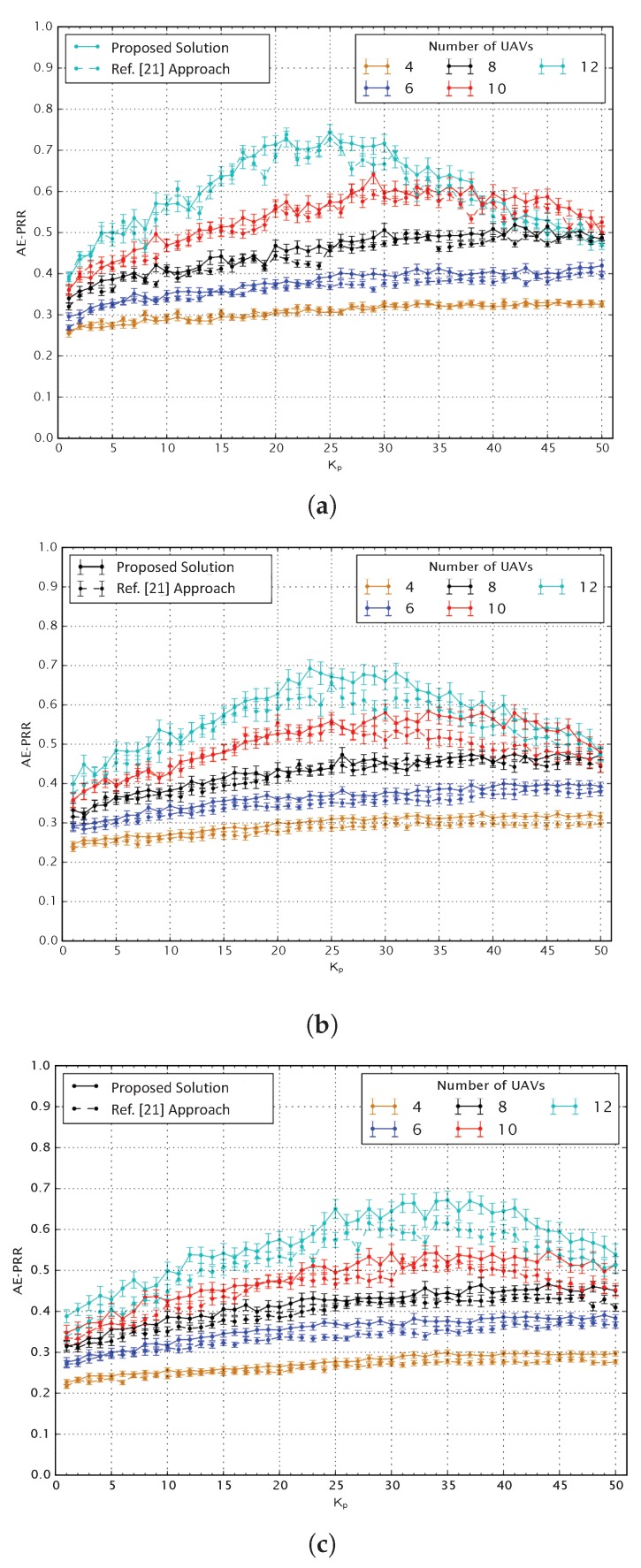
Average End-to-End Packet Reception Rate (AE-PRR) versus $K_{p}$ for (**a**) 3 teams of 20 GNs (**b**) 4 teams of 20 GNs (**c**) 5 teams of 20 GNs.

Figure 5 shows the Average End-to-End Packet Reception Rate (AE-PRR) as a function of different values of $K_{p}$. It is worth mentioning that the metric AE-PRR is the average percentage of unique packets sent by all GN and correctly received by the BS through the network. To evaluate the performance of the proposed solution, we compare our approach with that of [24]. In the figure, our proposed approach is shown as a solid line, whereas, the approach of [24] is illustrated as a dashed line. In general, it can be seen that the proposed approach outperforms the conventional approach of [24] in terms of improved AE-PRR. In addition to this, it can be noticed that the number of teams influences the range of $K_{p}$ values that gives the best results in terms of AE-PRR. As an example, with only 4 teams and 12 UAVs, the best results are obtained with $K_{p}$ in the range [20, 30], while with 5 teams and 12 UAVs the best range becomes [30, 40]. In general, we can observe that a bigger number of GNs requires bigger $K_{p}$ values. Another immediate and obvious consequence of adding more GNs is the decrease of the maximum achievable AE-PRR, which passes from around 0.9 with one team to almost 0.65 with five teams. Furthermore, one can observe that the difference between the packet reception rate of the proposed solution and that of [24] grows for a larger number of UAVs in the network. This is one of the most critical aspects of the proposed solution is that the high packet loss might be too penalizing for a critical application. Even when just 60 GNs divided into three teams are deployed and 12 UAVs are used, the packet loss is above 20%. This is the drive that brought to the development of the CRM and MP algorithms.

**Figure 6:**
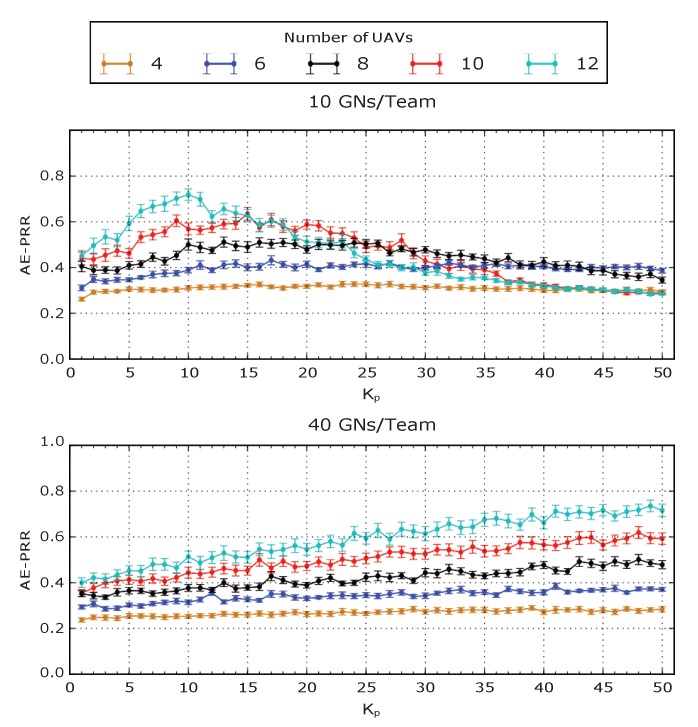
AE-PRR against $K_{p}$ with 3 teams composed of different numbers of GNs.

$K_{p}$ is not only influenced by the number of teams, but also by the number of GNs per team, as it is possible to observe in Figure 6. The figure shows the AE-PRR of two simulation scenarios: one having 3 teams of 10 GNs and the other having 3 teams of 40 GNs. Compared with the results reported in Figure 5, in the scenario with only 10 GNs per team, the system behaves better with a lower $K_{p}$ (range [5, 10]). On the contrary, in the scenario with 40 GNs per team, the system performs better with a higher $K_{p}$ (range [45, 50]). This shows that a higher density of GNs moving in the same area requires bigger values of $K_{p}$ to obtain the best AE-PRR results.

**Figure 7:**
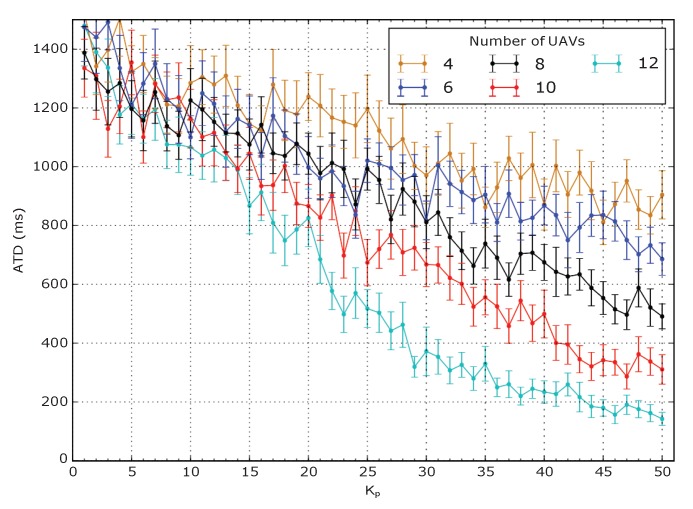
Illustration of Average Total Delay (ATD) with 3 teams of 20 GNs.

It is also interesting to study the trend of the Average Total Delay (ATD) which is expressed as the average delay of packets sent by GNs to the BS through UAVs. Specifically, it is the sum of the delay experienced in the LoRaWAN network segment and in the WiFi network segment while it also includes the delay caused by buffered packets. In this regard, a plot is shown in Figure 7 for three teams of GNs. The ATD decreases with the number of UAVs and with bigger values of $K_{p}$. To understand this behavior, it is necessary to remember that the ATD is computed only on packets that are successfully received by the BS and it takes into account also the time that a packet eventually spends in the buffer. Therefore, in scenarios with few UAVs, the mesh is more subject to disconnections and the bigger number of buffered packets contributes in large part to the big delay.

**Figure 8:**
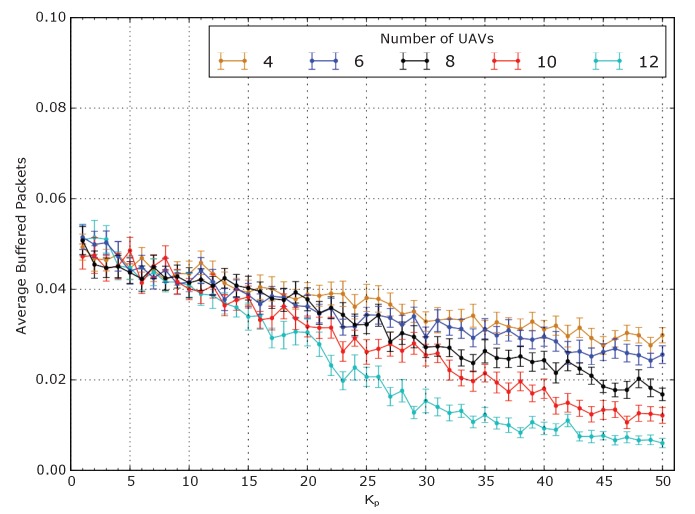
Average percentage of buffered packets in a scenario with 3 teams of 20 GNs.

Disconnections and buffering time also explains why lower delays are associated with bigger values of $K_{p}$: a big $K_{p}$ favors the compactness of the aerial mesh to the detriment of the coverage. This is confirmed by the decreasing trend of the average percentage of buffered packets shown in Figure 8, which is an indication of the probability of disconnections in the mesh. $K_{p}$ must, therefore, be chosen as a compromise between the desired coverage and delay. The analysis of the other QoS metrics produces some minor observations. In conclusion, $K_{p}$ proved to be an important parameter that greatly influences the performance of the system. It has, therefore, to be chosen properly depending on the number of UAVs, the number of teams and the number of GNs per team. Configuring $K_{p}$ might be complex in a real system, due to the numerous variables that must be taken into account. A future study of a $K_{p}$ optimization routine is, therefore, necessary to simplify the configuration process.

## 5. Conclusions

In this paper, a new network system called the UAV-enabled LoRa network has been designed. The main objective of the system is to extend the network coverage offered by a BS to a set of mobile GNs using relaying gateways installed on UAVs. This objective is achieved thanks to a double-layer network that consists of a LoRaWAN segment for the communication between the GNs and the gateways and a WiFi ad hoc network for all the communications between the gateways and the BS. The core of the system is the UAV-enabled LoRa network mobility algorithm, specifically designed to adapt as much as possible to the ever-changing position of the GNs. It is shown that the activation of all the UAV-enabled LoRa network algorithms (VSF + CRM + MP) allows us to obtain an AE-PRR above 0.9 in a representative scenario with three teams of GNs and 12 UAVs. An acceptable AE-PRR, between 0.8 and 0.9, is obtained with 8 or 10 UAVs. We anticipate that our work would be helpful in the practical realization of such disaster management networks.

Although the presented architecture shows promise, there are some additional features that can be explored to continue and possibly upgrade what was accomplished in the present work. For instance, UAVs are subject to strict energy constraints. The impact of such constraints is of great practical interest. The developed algorithm may need to be modified to limit as much as possible unnecessary movements and transmissions. A future iteration of the system might also consider the usage of fixed-wings drones to increase the maximum air-time. Moreover, the impact of outdated channel estimation can also be investigated for practical systems. These challenging tasks are left for future studies.

## Figures and Tables

**Table 1:** Comparison between the state-of-the-art and the proposed Unmanned Aerial Vehicle (UAV)-enabled Long Range (LoRA) network.

Reference	Ideology	Approach	LoRaWAN	Relay-Aided	Resilience	Wireless Support
[28]	Surveillance system based on the cognitive IoT	Amateur surveillance system	No	Yes	-	Yes
[33]	Multi-UAV surveillance	Motion planning	No	Yes	No	Yes
[34]	Multi-constraints in a 3D environment	UAV path planning	Yes	Yes	-	No
[35]	Network centric systems	Urban surveillance	No	Yes	-	Yes
[30]	LoRa wireless network	Meteorological information display	Yes	No	-	Yes
[36]	Low-cost, low-power and long-range visualization	Visual surveillance	Yes	Yes	-	Yes
[37]	Key points matching problem	Feature detection of nonconforming objects	No	Yes	No	No
This work	UAV-enabled LoRa network	Proposed approach	Yes	Yes	Yes	Yes

**Table 2:** Simulation parameters and their values.

Simulation Parameter	Value
Total simulation time	2000 s
Tx Power	14 dBm
Bandwidth	125 KHz
Packet Size	10 Bytes
GN Speed	1.3 m/s
$p_{shared}$	0.5
$t_{load}$	180 s
PHY protocol	IEEE 802.11 g
Frequency	2.4 GHz
Receiver sensitivity	−99 dBm
ΔT	10 s
$t_{AtG}$	40 s
$LB_{req}$	20 dBm
$P_{max}$	30
$k_{max}$	3
$s_{thr}$	0.6
UAV altitude	60 m
$t_{lost}$	180 s
$t_{r}$	40 s
